# The metabolic adaptation in wild vertebrates via omics approaches

**DOI:** 10.1093/lifemeta/loac040

**Published:** 2022-12-28

**Authors:** Xin Du, Yisi Hu, Guangping Huang, Fuwen Wei

**Affiliations:** Key Laboratory of Animal Ecology and Conservation Biology, Institute of Zoology, Chinese Academy of Sciences, Beijing 100101, China; University of Chinese Academy of Sciences, Beijing 100049, China; Key Laboratory of Animal Ecology and Conservation Biology, Institute of Zoology, Chinese Academy of Sciences, Beijing 100101, China; University of Chinese Academy of Sciences, Beijing 100049, China; Center for Evolution and Conservation Biology, Southern Marine Science and Engineering Guangdong Laboratory (Guangzhou), Guangzhou, Guangdong 511458, China; Key Laboratory of Animal Ecology and Conservation Biology, Institute of Zoology, Chinese Academy of Sciences, Beijing 100101, China; Key Laboratory of Animal Ecology and Conservation Biology, Institute of Zoology, Chinese Academy of Sciences, Beijing 100101, China; University of Chinese Academy of Sciences, Beijing 100049, China; Center for Evolution and Conservation Biology, Southern Marine Science and Engineering Guangdong Laboratory (Guangzhou), Guangzhou, Guangdong 511458, China

**Keywords:** adaptative evolution, metabolic adaptation, comparative genomics, multi-omics, epigenetics

## Abstract

Metabolism is the basis for sustaining life and essential to the adaptive evolution of organisms. With the development of high-throughput sequencing technology, genetic mechanisms of adaptive evolution, including metabolic adaptation, have been extensively resolved by omics approaches, but a deep understanding of genetic and epigenetic metabolic adaptation is still lacking. Exploring metabolic adaptations from genetic and epigenetic perspectives in wild vertebrates is vital to understanding species evolution, especially for the early stages of adaptative evolution. Herein, we summarize the advances in our understanding of metabolic adaptations via omics approaches in wild vertebrates based on three types of cases: extreme environment, periodically changing environment, and changes of species characteristics. We conclude that the understanding of the formation of metabolic adaptations at the genetic level alone can well identify the adaptive genetic variation that has developed during evolution, but cannot resolve the potential impact of metabolic adaptations on the adaptative evolution in the future. Thus, it seems imperative to include epigenomics and metabolomics in the study of adaptation, and that in the future genomic and epigenetic data should be integrated to understand the formation of metabolic adaptation of wild vertebrate organisms.

## Introduction

Metabolism is the basis for life-sustaining activities, and is also a complex network of biochemical reactions catalyzed by enzymes that regulate the concentration and reaction rate of substrates and products [1]. Life depends on adapting to changing environments, but life activities require metabolism to maintain homeostasis, so organisms need to constantly balance the effects of changing environments with their bodily needs to maintain survival and reproduction. The most direct manifestation of the interplay between the internal and external environment is the change in metabolism. The changes of matter and energy in the environment cause the metabolic state of the living organism to change [1], but the organism will eventually form a metabolic state that adapts or acclimatizes to it through a series of regulations. We defined the change in metabolic states that occurs in response to environmental perturbations or other changes as metabolic adaptation (MA). It is well known that genetic variants are the basis of adaptative evolution, and epigenetic variants (mainly including DNA methylation, histone modifications, and non-coding RNAs) are not only affected by metabolites, but also involved in the regulation of metabolic processes. Therefore, we believe that it is important to understand the MA from a genetic and epigenetic perspective. Further, the MA in this review can be either MAs developed during evolution [i.e. genetic code alterations, referred to as genetic metabolic adaptation (GMA)], such as genetic signals of MAs in high-altitude adaptation in Tibetans [2], or temporal adaptive changes in response to environmental disturbance [i.e. epigenetic changes of adaptation, referred to as epigenetic metabolic adaptation (EMA)], such as EMA of cancer cells to hypoxia [3].

**Figure 1:**
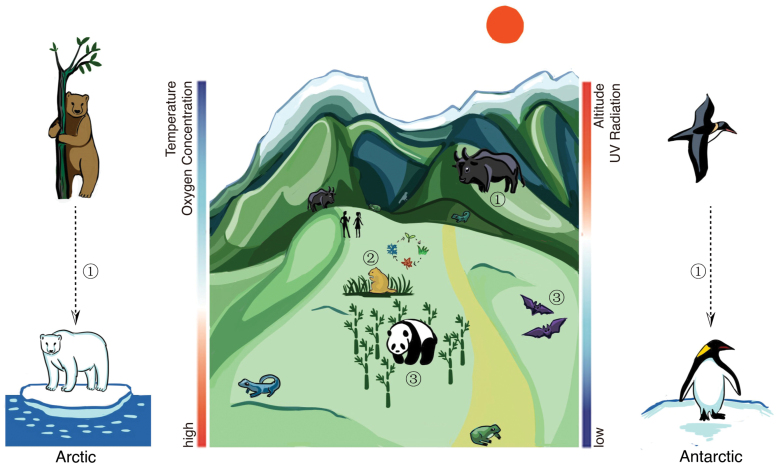
The diagram of three types of MAs. The numbers in the circles correspond to the three types of MAs described in this review. ① MAs to extreme environments; ② MAs to periodic changes in the environment; and ③ MAs to changes in a species’ own characteristics. The gradient color bars indicate changes in oxygen concentration, temperature, UV radiation intensity, and elevation, where the red indicates high values and blue indicates low values.

Vertebrates are highly diverse and occupy a wide variety of ecological niches, which have provided several interesting cases of MAs during their long evolutionary history. With the emergence and rapid development of high-throughput sequencing technology, a multitude of wild vertebrate genomes have been sequenced, and the study of MAs as part of adaptive evolution has entered the genomic era. By the end of 2021, ~1770 vertebrate genomes have been assembled [4], which provide an important genomic resource for comprehensive and in-depth comparative genomics studies. Over the past decade, multi-omics technologies have also been developed, and many cases of multi-omics research combining genomics with comparative genomics have emerged [5–8], helping out understanding of the MAs by wild vertebrates. Herein, we summarize three types of MAs, including: (i) MAs to extreme environments, such as the MA in polar and high-elevation environments; (ii) MAs to periodic changes in the environment, such as the hibernation in mammals; and (iii) MAs to changes in a species’ own characteristics, such as giant and red pandas or bats (Fig. 1). We hope that this review will also provide an outlook on the application of epigenomics and metabolomics in the adaptive evolution of wild vertebrates and new insights into the formation of adaptative evolution in the future.

## MA to extreme environments

### MA to the polar environments

Polar environments are characterized by polar day and night, extreme cold, lack of fresh water, and extreme food scarcity [9]. Penguins (Sphenisciformes) and polar bears (*Ursus maritimus*), as the representative species of the South and North Poles, respectively, have developed many adaptations to these extreme environments. Earlier studies have shed light on their adaptive evolutionary mechanisms in terms of physiology and body structures, such as abundant adipopexis under the skin and around the organs, as well as prolonged seasonal fasts, in polar bears to cope with severe cold and food deprivation [10, 11], and the scaly plumage of penguins that repels water and insulates against heat [12, 13]. Some reviews have summarized the behavioral, physical, and physiological means by which mammals and birds adapt to polar environments through maintenance of body temperature stability and the reduction of energy expenditure, but little is known about the underlying genetic adaptation [9].

To uncover the genetic basis by which polar bears have adapted to the arctic environment, Liu *et al*. identified 9 of the top 16 genes under selection based on resequencing data that were associated with cardiomyopathy and vascular disease, with the strongest selection signal detected for the *APOB* gene encoding the primary lipoprotein component of low-density lipoprotein [14]. Furthermore, studies based on copy number variation showed that several genes related to fatty acid metabolism had significantly fewer copy numbers in the polar bear genome than that in the brown bears [15]. Notably, the emergence of these GMAs is inextricably linked to a high-fat diet and polar life in polar bears. Unfortunately, much remains unclear about the GMAs of polar bears to the polar environment, such as how they cope with freshwater scarcity, how they use fat efficiently, how they ­conserve energy, and how they survive chronic food deprivation [16, 17]. However, it cannot be ignored that polar bears are now at certain risk of extinction [18, 19], and hopefully there is still an opportunity for humans to continue mining valuable information on the GMAs in this species and to apply it in their conservation.

To unravel the genetic basis of adaptation by penguins to the Antarctic climate, Li *et al*. conducted genomic and comparative genomic analyses and found that the *FASN* gene, which encodes a fatty acid synthase and participates in lipogenesis, was positively selected, and that four lipid metabolism-related genes were under positive selection in the ancestral branches of Adélie penguins (*Pygoscelis adeliae*) and Emperor penguins (*Aptenodytes forsteri*) [20]. Furthermore, based on a larger genomic dataset, four genes related to thermoregulation were positively selected in the common ancestral nodes of the penguin [21]. Interestingly, penguins need to reserve sufficient blood oxygen and utilize anaerobic respiration for prolonged underwater foraging activities [22–24], and a large-scale genome comparison identified seven genes associated with oxidation that were positively selected or had penguin-specific sites substitutions [20]. Notably, comparative genomics studies of both the Arctic fox (*Vulpes lagopus*) [25] and the reindeer (*Rangifer tarandus*) [26] also identified genes of adaptative evolution related to lipid metabolism, hinting a possible functional convergent evolution of polar terrestrial vertebrates, but this speculation needs more studies of comparative genomics of polar species to support it.

It can been seen from above cases of MAs, the GMA plays a key role in the adaptation of the polar environment, and the adaptations in body structure, physiology, and behavior are all centered on maintaining a stable metabolic state. However, the metabolic and epigenetic states of wild vertebrates in polar environments are rarely reported, and perhaps further combining epigenetic and metabolic data could enhance our understanding of the formation of polar MAs.

### MA to high-elevation environments

High-elevation environments are characterized by lower atmospheric oxygen content, higher levels of UV radiation, and reduced temperature compared with low-elevation environments, which pose a challenge to the survival of many species [27]. One of the typical representatives of high-elevation regions is the Qinghai-Tibetan Plateau (QTP), which has a unique and rich biodiversity and is one of the global biodiversity hotspots, as well as the most ideal area to study the adaptive evolution to high-altitude environments.

To understand the genetic basis of high-elevation adaptations, the domestic yak (*Bos grunniens*), one of the representative large mammals of the Tibetan plateau, was sequenced, and a significant enrichment of positive selection genes to hypoxia response and energy metabolism was found by comparative genomic analysis [28]. Five genes under positive selection in yaks, *Hsd17b12*, *Gcnt3*, *Camk2b*, *Whcs1*, and *Glul*, were involved in nutritional metabolic processes, such as lipid metabolism, gluconeogenesis, and amino acids [28]. Subsequently, a study integrating single-cell transcriptomics and genome structural variation data revealed a new subtype of endothelial cells and identified a series of genes and pathways associated with lung development, which provided new insights into the plateau adaptation by the yak [7]. Furthermore, in the ground tit (*Parus humilis*), it was also found that genes related to hypoxia response and fatty acid metabolism are positively selected, and the gene families related to energy metabolism have expanded to cope with the challenge of chronic cold stress conditions [29]. Interestingly, the positively selected genes related to hypoxia response and energy metabolism have many overlaps between the yak and the ground tit, such as *HIF1AN*, *ANGP4*, *ADAM9*, and *MDH1B*, which are genes associated with the hypoxic response [29]. Specifically, HIF1AN is an inhibitor of HIF-1α, ANGP4 is speculated to possibly facilitate systemic transport of oxygen, ADAM9 is an important regulator of HIF-1α, and MDH1B is predicted to be possibly involved in the tricarboxylic acid cycle. This suggests to us that genes not directly involved in metabolic processes are also part of GMA, for instance, *ANGP4*. Further studies of different species of birds living at high altitudes show a diversity of genetic locus variation, but a high degree of functional homogeneity, i.e. parallel functional evolution of hypoxia and cold responses, has occurred [30]. This parallel functional evolution has also been confirmed in studies of adaptation to the QTP in amphibians and reptiles [27]. In addition, many other cases of plateau adaptation studies have also detected adaptive selection signals in the hypoxia response, low temperature tolerance, and DNA damage repair [8, 31–33], which may indicate a possible functional convergence in GMAs in vertebrates living on the Tibetan plateau.

Notably, most studies have focused on species that have produced phenotypically stable adaptations, and little has been reported about short-term acclimation and acclimatization. For example, genomic, population genomic, and transcriptomic findings of Eurasian tree sparrows (*Passer montanus*) confirmed that 1-month rearing under plateau conditions did not cause changes in muscle structure in this species [34], but this work did not take their epigenetic and metabolic level of alterations into account. Next, the same research team combined transcriptomic data through cellular experiments, and found that primary fibroblasts from great tits and mice stimulated by short-term simulated high-altitude conditions (i.e. low oxygen) also exhibit metabolic traits adapted to high-altitude environments [35]. Furthermore, the associated differentially expressed genes (DEGs) were similar to those found in previous studies that underwent metabolic adaptive evolution, suggesting that phenotypic plasticity might influence or even promote adaptive evolution in the long-term [35], and certainly GMAs are of no exception. Excitedly, a study that combined population genomic, comparative genomic, and multi-omics analyses of the saker falcon has systematically revealed key roles of gene flow and non-coding regulatory regions in the GMAs of high-altitude environments, which supports the notion that the combination of these omics technologies can help better understand GMA mechanisms [6]. Interestingly, in Tibetans, some genetic signals are associated with altered metabolic states [36–38]; for instance, mutations in *EPAS1* are related to enhanced glycolysis, while mutations in *PPARA* are related with a reduced ability for fatty acid oxidation.

Overall, these cases of adaptive evolution at the QTP have focused on the hypoxic response, energy metabolic balance, UV radiation resistance, and phenotypic adaptation, revealing the key role that GMA plays in long-term adaptation, while showing the importance of GMAs in the evolution of high-elevation adaptations.

## Wild vertebrates adapt to periodically changing environments

### MA of hibernation in mammals

The Earth’s revolution around the sun produces four seasons, which creates distinct seasonal environments of temperature and food resources at mid-latitude region. Some mammals will take the initiative to lower their body temperature throughout the winter to conserve energy and improve survival—a state known as hibernation (or multiday torpor) [39]. Early studies on the adaptation of mammalian hibernation focused on the characterization of the altered physiological properties associated with it, and its neural regulation [40–42] and after 1992 the molecular adaptation mechanisms of hibernation were reported [43–46].

In the early stages of the development of omics technology, the molecular mechanisms of hibernation in mammals benefited from findings of the transcriptome and proteome, and it was found that DEGs were central to hibernation in mammals [47]. Subsequently, many genes specifically up-regulated during hibernation were found by comparative transcriptomic analyses of different organs, tissues, and species, e.g. *PDK4* (pyruvate dehydrogenase kinase isoenzyme 4) [45], *ATF4* (activating transcription factor) [48], *HIF-1* (hypoxia inducible factor) [49], and *PPARγ* (peroxisome proliferator-activated receptor γ) [50] are up-regulated during hibernation. Furthermore, a study of the metabolome of 13-lined ground squirrels (*Ictidomys tridecemlineatus*) during hibernation found that hydrogen sulfide (H_2_S) may play an important role in the cyclic regulation of hibernation and the protection from physiological damage during hibernation [51]. Recently, the neural mechanisms of hibernation in mice were successfully elucidated via integrating single-nucleus RNA-sequencing and neural science experiments, and a hibernation-like state had been artificially induced in mice [52, 53]. In addition, a study on gut microbes in *I. tridecemlineatus* has shown that gut microbes are critical for host nitrogen metabolic homeostasis during hibernation [54]. Obviously, differential gene expression, neuromodulation, and gut microbial adaptation all play important roles in MA during hibernation, and this may be less dependent on genetic variation in the genomes of hibernating species.

It is important to note that hibernation-related genes are mostly present in non-hibernating species, but only hibernating species can spontaneously induce regulation of their programmed expression. However, few genetic mechanisms for hibernation-specific MAs have been reported based on the available studies. An earlier comparative genomic article reported that *Leptin* underwent adaptive evolution in the ancestral nodes of bats [55], but later work refuted this conclusion [56]. Intriguingly, the population genome of *I. tridecemlineatus* identified genetic loci associated with the seasonal onset of hibernation, especially a putative causal variant in the promoter of *FAM204A*, which is potentially related with a response to the onset of fasting and a switch to fatty acid metabolism [57, 58]. Moreover, a recent study based on functional genomics found that the *PER2* (period circadian regulator 2) gene in the loris genome was positively selected, which is potentially associated with the metabolic regulation to hibernation [59].

In conclusion, hibernation is one of the seasonal change adaptations, and MAs like this to periodic perturbations of the environment do not necessarily need to arise by genetic changes in coding regions, but may instead occur more as the result of synergistic regulatory effects of humoral regulation, neuromodulation [60], and epigenetic changes, including non-coding regulatory regions [43].

## MA for changes of a species’ own characteristics

### MA of diet shift in giant panda

The giant panda (*Ailuropoda melanoleuca*) has evolved unique morphological and physiological phenotypes to adapt to changing environments during the last 7–8 million years [61]. The bamboo-eating diet is the most representative adaptive trait in giant pandas, and numerous studies have centered around it, including exploring its effects on the metabolic rate, body structure, and gut microbial adaptations [62–66].

Since the publication of the next-generation sequenced genome of giant pandas, it has become possible to decipher the MA of giant pandas to bamboo-eating at the genetic level [67]. Based on the prediction of coding genes in the genome, it was found that the giant panda genome does not contain cellulose-digesting genes, and thus digestion of bamboo may mainly rely on gut microbes [67]. It is known that giant pandas need to consume a large amount of fresh bamboo or bamboo shoots every day, and bamboo accounts for about 99% of the diet of wild giant pandas [68], but its digestive utilization of bamboo dry matter is <20% [69]. Coincidentally, the red panda (*Ailurus fulgens*), which belongs to the family Ailuridae within the Carnivora [70], also has evolved a specialized bamboo diet. Intriguingly, genes involved in the digestion and utilization of bamboo nutrients (e.g. essential amino acids, fatty acids, and vitamins) that underwent adaptive convergence were identified by identical amino acid substitutions in the same gene in giant and red pandas [63]. Certainly, the gut microbes of giant pandas are also involved in the digestion of bamboo nutrients and even play a key role in responding to seasonal changes in foraging resources [64, 65, 71]. Besides, bamboo also contains a large number of secondary metabolites, and a study integrating multi-omics and metagenomics found that the content and kinds of flavonoids in bamboo have positive effects on the gut microbial structure and cellulose digestion of giant pandas [72]. By comparative genomics of giant pandas and red pandas, a research also found that the adaptive evolution of the *TAS2R* gene family may be related to the selection of bamboo species to avoid consuming toxic substances [73]. Similarly, the koala-specific duplications of *TAS2R* gene family were found in four marsupial orthologous groups that enable recognition of hypotoxic eucalyptus leaves [74].

However, low-calorie intake and the low digestibility of bamboo still pose a challenge to the survival of the giant pandas, and thus it needs a low-energy metabolism to adapt to its diet and to maintain its survival and reproductive capacity. The integration of multidisciplinary techniques, such as double-labeled water methods, genomics, and thermal imaging technologies, confirmed that the giant panda has a low-metabolic rate [66]. Furthermore, the approach identified that the dual-oxidase 2 (*DUOX2*), a gene critical for thyroid hormone synthesis, is specifically mutated in giant pandas, suggesting that it may be associated with low-energy metabolism in the species [66]. In a later study, genome-edited mice confirmed that the single-nucleotide mutation of *DUOX2* in giant pandas is key to the formation of the giant panda’s metabolic phenotype [75]. Intriguingly, the organ sizes of the brain, liver, and kidneys are all >10% smaller than expected in giant pandas, which is also key to lower energy metabolism [66]. The researchers speculated that this may be associated with the adaptive evolution of Hippo pathway genes or related regulatory regions [76, 77].

Overall, the GMAs of the bamboo-eating giant panda do not only occur in energy metabolism, but also involve the synergistic participation of body structure, digestion and absorption, gut microbiota, behavioral adaptations, and organ size reduction, suggesting to some extent that the MAs of bamboo-eating giant pandas are a typical case of systematic adaptive evolution [62].

### MA of flight in bats

The bat (Chiroptera) group has >1400 species [78], with many unique adaptive traits, including the ability to fly, which is the most unique among mammals, and its adaptation to flight has been of great interest to evolutionary biologists. It is well known that flight is one of the most energy-intensive forms of locomotion, and bats must be able to sustain such a large energy expenditure [79, 80], which means that the MA of bats to flight is very different from the previous case of energy conservation.

To figure out the genetic basis of high-metabolic rate, a comparative genomic analysis revealed that positive selection signals were detected in 23% of mitochondrial-encoded genes and 4.9% of nuclear-encoded genes along the oxidative phosphorylation (OXPHOS) pathway, speculating that the adaptive evolution of the OXPHOS pathway may be key to the adaptation of bats to flight energy metabolism [81]. Elevated metabolic rates via the OXPHOS pathway provide a strong energetic support for flight, but are subsequently confronted with problems of energy balance and cellular damage associated with high-metabolic rates. In fact, bats have been well adapted to the high-energy demands of flight, which means that bats are characterized by highly metabolic stability [82], and thus the maintenance of this stability requires specific GMAs. Draft genomes of two bat species revealed the mechanism of GMA for bat flight by comparative genomic means, namely, multiple genes in the DNA damage checkpoint and nuclear factor-κB pathway at ancestral nodes were subjected to positive selection, which may have balanced the high-metabolic rate with protection from genomic damage [83]. Notably, the maintenance of high-metabolic rate is dependent on the supply of enough fuel, as the limitation of flight does not allow taking large amounts of food to gain excess weight or over-storing fat to increase the flight burden, and this balance of material and energy requires that bats themselves produce many GMAs. Some studies have revealed that bats have efficient nutrient digestion and absorption from the perspective of intestinal absorption efficiency [84–86]. Moreover, the gut microbiota also plays a crucial role in the MA of bats for flight [87]. It is worth noting that a study on how fruit-eating bats (*Uroderma bilobatum*) balance the energy expenditure of taking fig juice observed a series of unexpected results. Notably, it was found that these bats can offset the energy expenditure from flight foraging by intermittently lowering their heart rate, and in addition, they can rapidly absorb nutrients from food and use them directly for flight energy, and surprisingly, these bats can exchange fat reserves within 24 h [88]. Unfortunately, although the bat genomes have been extensively resolved [89–91], the genetic mechanisms of high-efficiency fuel storage and utilization and the huge metabolic rate fluctuations have not been resolved, which may require further integration of the glycome, lipidome, metabolome, and epigenome to address the genetic mechanism of material MA.

In conclusion, the adaptive evolution of the OXPHOS pathway is the key GMA for the MA of bat flight, and the gut microbes, efficient digestive system, and well-developed DNA damage repair mechanism are important secondary mediators of the adaptation. Certainly, the genetic mechanisms of the corresponding material metabolism, gastrointestinal digestion, and heart rate modulation are still unclear and may need to be resolved by combining more omics data and downstream experimental validation.

## Conclusions

Metabolism is fundamental to the maintenance of life activities, and the development of GMAs is essential for organisms to adapt to specific environments in evolutionary processes. Omics-based approaches provide powerful methodological support for deciphering specific GMAs, as best evidenced by the three types of MAs highlighted in this review. In fact, there are extremely rich cases of MA in nature, such as the second aquatic adaptations of vertebrates, koala eating eucalyptus leaves, vertebrates living in desert areas, and so on. However, precisely because of the importance of metabolism, it is relatively difficult for metabolism-related core coding genes to undergo substantial genetic variation, and we maybe ignore the role of EMAs in wild vertebrates. Notably, there are limitations in explaining the GMA of hibernation and short-term adaptation based only on the genetic level, hinting that we should try more types of methods and data to answer related questions of EMAs.

**Figure 2:**
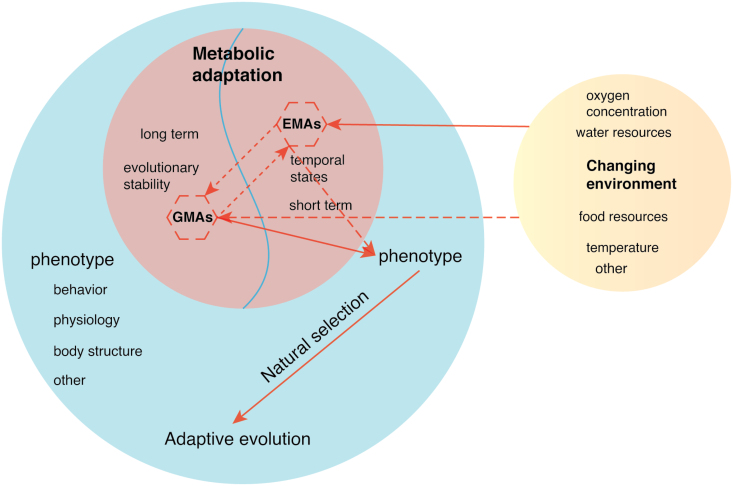
Schematic of MAs driven by the environment in wild vertebrates. The dotted lines indicate weak and doubtful connections, and the solid lines represent strong and real connections.

Interestingly, in two studies on hibernation and aging in big brown bats and yellow-bellied marmots, hibernation was found to delay epigenetic aging [92, 93], which aptly suggests that metabolism and epigenetics are inextricably linked. It is worth mentioning that a recent study on the epigenetic adaptation of a Mexican fish, *Poecilia mexicana*, to H_2_S-rich springs found that genes in differentially methylated regions were enriched to sulfur toxicity and sulfur metabolism [94]. Nevertheless, the authors mainly focused on the fact that this methylation pattern can be inherited [94], without noticing that this is an adaptation formed by the combined action of metabolism and epigenetics. These preliminary studies in wild vertebrates suggested that the method of integrating metabolism and epigenetics is a powerful tool for resolving the mechanism underlying MAs. It is evident that considering both metabolic and epigenetic and even other omics information in the study of MAs could provide new insight into early adaptive evolution, short-term stress response mechanisms, and complex phenotypical formation. Further, the variants of epigenetic modification are rapid responses to a changing environment, and this response may further influence the occurrence of DNA variants, such as 5-methylcytosine (5mC), possibly through hydrolytic deamination to thymine [95], so we hypothesized that EMAs could influence GMAs in response to a changing environment during the early formation of MAs, and even affect the phenotype of individuals and thus be fixed by natural selection. It could even provide strong evidence for a unified theory of the molecular aspects of evolution [96]. Therefore, we propose that it is possible that the environment drives the EMA and thus affects the GMA (Fig. 2).

## Perspective on integrated metabolic and epigenetic multi-omics research

We are now in the era of high-throughput sequencing, where we have an increasing wealth of approaches to solve traditional problems. Most of the cases cited in this review have benefited from the development of omics technologies to be able to genetically resolve the mechanisms of MAs in large numbers. In other words, genomic, comparative genomic, and population genetic techniques, which mainly address evolutionary questions, are already well suited to address the need for genetic resolution of MAs. However, the application of these technical tools alone can hardly be used to address scientific questions that are ongoing or in which genetics is not primarily involved (e.g. EMA and state turn-on in hibernation), or to answer the question of whether short-term MAs can affect evolution. In addition, many wild vertebrates are now facing many threats because of multiple problems, such as global warming, and thus there is a need to further understand how these animals are responding to this crisis. It is worth noting that there have been many studies utilizing the combination of metabolism and epigenetics in humans and mice that primarily focused on the shaping of nutrient, alcohol, and microbial metabolism on states of metabolism and epigenetics, with a special focus on human metabolic diseases [97–104]. In other words, epigenetic and metabolic studies in humans and mice could provide important references and lessons for studies of MAs in wild vertebrates. Hence, a combined metabolic and epigenetic approach may be effective in addressing these challenges in the study of MAs.

In conclusion, EMA studies are currently extremely scarce, and we hereby advocate for more multi-omics studies integrating metabolism and epigenetics in wild vertebrates to provide new insights into early adaptive evolution, endangered species conservation, and the development of complex traits in wild vertebrates. In addition, if plentiful related studies are conducted in the future, we can draw insight into the mechanisms of phenotype shaping by environment–gene interactions from the vast biological treasury of nature, and gain more insight into how such interactions synergistically shape phenotypes or cause complex diseases in humans.
